# Ventilation Targets for Patients Undergoing Mechanical Thrombectomy for Acute Ischemic Stroke: A Systematic Review

**DOI:** 10.3390/jcm12154925

**Published:** 2023-07-26

**Authors:** Alessandro Scudellari, Paula Dudek, Luca Marino, Rafael Badenes, Federico Bilotta

**Affiliations:** 1 Anaesthetic Department, Addenbrooke’s Hospital, Cambridge CB2 0QQ, UK; 2 2nd Department of Anesthesiology and Intensive Care, Medical University of Warsaw, 02-097 Warsaw, Poland; paoladudek@gmail.com; 3 Department of Mechanical and Aerospace Engineering, “Sapienza” University of Rome, 00184 Rome, Italy; 4 Department of Anesthesiology and Surgical-Trauma Intensive Care, Hospital Clínic Universitari de Valencia, University of Valencia, 46010 Valencia, Spain; 5 Department of Anesthesiology, Critical Care and Pain Medicine, Policlinico Umberto I, “Sapienza” University of Rome, 00185 Rome, Italy; federico.bilotta@uniroma1.it

**Keywords:** mechanical thrombectomy, acute ischemic stroke, mechanical ventilation, oxygenation, partial pressure of carbon dioxide, end tidal carbon dioxide, general anesthetic, pO_2_, pCO_2_, ETCO_2_

## Abstract

Mechanical thrombectomy (MT) has become a standard treatment for acute ischemic stroke (AIS) caused by large vessel occlusion (LVO). Recent evidence suggests that general anesthesia (GA) and mechanical ventilation do not lead to inferior neurologic outcomes if compared to non-GA. However, the guidelines lack specific recommendations for ventilation targets during MT under GA. This systematic review aims to identify ventilation strategies correlating with better neurological outcomes in AIS patients undergoing MT, particularly focusing on oxygenation and carbon dioxide (CO_2_) targets. A systematic search of multiple databases was conducted to identify human studies reporting the correlation between ventilation strategies and neurological outcomes in MT for AIS. Eligible studies included clinical trials, observational studies, and case–control studies. Out of 157 studies assessed, 11 met the inclusion criteria. Five studies investigated oxygenation targets, while six studies explored CO_2_ targets. The published studies highlighted the controversial role of supplemental normobaric oxygen therapy and its potential association with worse outcomes. Regarding CO_2_ targets, the studies identified a potential association between end tidal CO_2_ levels and functional outcomes, with hypocapnia being unfavorable. This systematic review demonstrates that the current available evidence still lacks strength to suggest specific ventilation targets, but it highlights the potential risks of hyperoxia and hypocapnia in this specific cohort of patients.

## 1. Introduction

Acute ischemic stroke (AIS) is a leading cause of death and disability worldwide [1]. In 2019, there were 12.2 million incident strokes and 6.55 million stroke-related deaths, making stroke the second-leading cause of death globally, after ischemic heart disease. Ischemic strokes account for approximately 85% of all cases. The prompt restoration of blood flow is crucial for salvaging the surrounding brain tissue in the penumbral area, with the extent of damage depending on the location of the blockage, local collateral circulation, and the time taken for blood flow to resume. Recanalization techniques such as thrombolysis or mechanical thrombectomy (MT) are used to restore blood flow. While thrombolysis efficacy for large vessel occlusion (LVO) is limited, MT has emerged as a standard treatment for LVO [2]. The role of general anesthesia (GA) during MT has been widely debated due to its potential advantages in facilitating operating conditions but also the concerns about additional risks such as increased time, hypotension, and hypoperfusion of the ischemic penumbra. Early observational studies suggested worse neurological outcomes and higher mortality with GA, but subsequent small randomized controlled trials (RCTs) indicated that GA may lead to better functional recovery and higher rates of reperfusion. The differences in results between earlier retrospective studies and recent prospective trials may be attributed to improved protocolization and strict anesthesia management in the latter. In 2022, a Cochrane meta-analysis pooled data from seven RCTs (involving 978 patients) investigating the correlation between GA and non-GA techniques in neurologic outcomes following MT for AIS. The analysis concluded that GA during MT does not result in inferior neurologic outcomes and may even favor better operating conditions and improved target artery revascularization. This represents the largest cohort of patients currently available, and while the sample size is relatively small, it supports the trend of a non-correlation between the chosen anesthesia technique and neurologic outcome, provided that the physiological parameters are tightly controlled by a specialized team [3]. The inclusion criteria for MT are rapidly evolving, extending both the time windows and vessel targets. Particularly, evidence is accumulating regarding the value of performing MT on more distal and smaller vessels, which necessitates higher precision in endovascular navigation and consequently requires patient immobility [4]. Consequently, it is highly likely that the number of patients undergoing MT under GA and controlled ventilation will increase.

The most recent guidelines for the management of patients with AIS published in 2018 and updated in 2019 suggest administering supplemental oxygen only when the peripheral oxygen saturation (SpO_2_) is less than 94% and to aim for normocapnia regarding the ventilation goals for these patients [5]. However, there is no specific guidance on how to manage the ventilation targets for patients undergoing MT for AIS under GA.

This systematic review (SR) aims to investigate if there are specific ventilation strategies that correlate with better neurological outcomes in patients undergoing MT for AIS; in particular, our search aims to identify specific targets of arterial oxygenation and arterial carbon dioxide partial pressure that correlate with better neurological outcomes.

## 2. Materials and Methods

The present SR was carried out according to the Preferred Reporting and Items for SR and Meta Analyses (PRISMA). The analysis was recorded in the PROSPERO registry database for systematic reviews (N. CRD42023405708 on 20 March 2023).

### 2.1. Eligibility and Study Selection

Human studies, clinical trials, observational studies, and case–control studies that reported on the correlation between ventilation strategy and neurological outcomes in patients undergoing MT for acute ischemic stroke were eligible. The primary outcomes were the correlation between arterial oxygenation and carbon dioxide partial pressure targets and neurological outcomes.

### 2.2. Search Strategy and Data Extraction

A systematic search of the PubMed, EMBASE, Cochrane Library, Clinicaltrials.gov, and WHO ICTRP databases was performed from inception until March 2023. 

The search strategy included the following keywords: stroke, cerebrovascular accident, mechanical thrombectomy, ventilation, artificial respiration, pO_2_, pCO_2_, partial pressure of (CO_2_ or O_2_, known as oxygen or carbon dioxide), end tidal CO_2_, and end tidal carbon dioxide (EtCO_2_). The screening was extended to the references of the selected studies.

**Figure 1:**
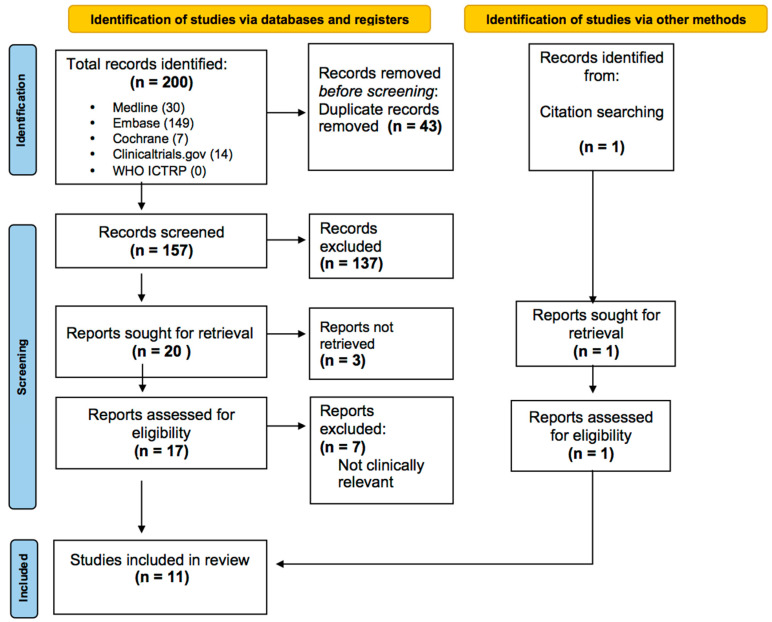
Prisma diagram.

All the results were uploaded on the Rayyan web system and independently assessed by 2 authors. When the screening process was completed, the results were un-blinded, and conflicts were resolved through discussion and mediation with a third author (Figure 1).

## 3. Results

Our search identified 200 records, and after the removal of duplicates, we assessed 157 abstracts. One hundred and thirty-seven were excluded, because they did not meet the inclusion criteria. The remaining 20 records were sought for retrieval, but only 17 were available, because 3 were only abstracts submitted to meetings. Of the 17 studies assessed for eligibility, 8 were excluded, because they were not clinically relevant. A total of 11 studies met the inclusion criteria. Five studies investigated the correlation of specific oxygenation targets and neurological outcomes. Six studies investigated the correlations of specific carbon dioxide targets and neurological outcomes.

### 3.1. Oxygenation Targets

Although, in this systematic search, there were not any identified studies that addressed the intraprocedural oxygenation targets for patients undergoing MT for AIS, we came across two published studies that focused their experiments on the early postoperative oxygenation targets in this specific population. We also identified three ongoing studies that were specifically relevant to these intraprocedural goals.

#### 3.1.1. Published Studies

- López et al., 2019 [6]

The study by López et al. aimed to investigate the effect of hyperoxia on functional recovery in patients with AIS who underwent MT. The study was a prospective observational cohort study that included all adult patients consecutively admitted to the intensive care unit (ICU) due to an AIS in the anterior cerebral circulation and following MT intervention between 2010 and 2015. There were 333 included patients. All patients were intubated and ventilated, receiving supplementary oxygen to achieve saturation above 94%. Two arms were defined based on the results of the first ICU blood gas analysis: one group with paO_2_ greater than 120 mmHg and one group with paO_2_ equal to or lower than 120 mmHg. The study found that high levels of paO_2_ were mostly related to worse functional outcomes, as measured by the modified Rankin Scale (mRS), after 90 days. The hyperoxia group had a significantly higher proportion of patients with poor functional outcomes compared to the paO_2_ ≤ 120 mmHg group. Mortality was also higher in the hyperoxia group. The study concluded that admission with paO_2_ > 120 mmHg was associated with worse functional outcomes in patients with AIS who underwent MT. This study had several limitations. Firstly, it was an observational study. Additionally, the supplemental oxygen administered until the start of MT was heterogeneous, and the study only had a single ICU admission blood gas analysis, so it was impossible to know for exactly how long the hyperoxia conditions were maintained [6]. The FiO_2_ provided during the MT was arbitrary, which could have affected the study’s results.

- Cheng et al., 2020 [7]

This was a prospective randomized controlled trial (RCT) conducted in a single center. The aim of the study was to evaluate the efficacy of supplemental high flow normobaric hyperoxia (NBO) for 6 h on functional outcomes in patients successfully treated with MT for AIS due to LVO in the anterior circulation. The NBO group was defined as having oxygen delivered by a Venturi mask with a fraction of inspired oxygen (FiO_2_) of 50% at a flow of 15 L/min. The control group received oxygen via nasal cannula at 3 L/min. A total of 175 patients were included in the final analysis (88 in the NBO group and 87 in the control group). The primary outcome was the mRS score at 90 days. The study found that NBO treatment resulted in a higher proportion of patients with improved functional outcomes, with an adjusted common odds ratio (indicating the odds of improvement of one point on the mRS) of 2.2 (95% CI, 1.26 to 3.87), favoring the distribution of global disability scores on the mRS at 90 days (*p* = 0.006) [7]. The study had several limitations, including that it was conducted in a single center, the absence of adjusted infarct volumes based on baseline data, and that the oxygen concentration in the blood was not monitored before or during the NBO process.

#### 3.1.2. Ongoing Studies

- NBOL (NCT05039697) [8]

Normobaric Hyperoxia Combined With Endovascular Therapy in Patients With Stroke Within 6 Hours of Onset: Long-term Outcome Analysis (NBOL) [8]. This interventional clinical trial aims to investigate the long-term outcomes of combining NBO therapy with endovascular mechanical thrombectomy in stroke patients within 6 h of onset. The NBO therapy involves delivering high-flow oxygen through an oxygen storage mask for 4 h, starting immediately after randomization. The trial aims to enroll 280 participants who will be randomized into either the NBO group or the control group, with the primary purpose of treatment. The estimated study completion date is 1 February 2023. The study’s experimental arm will receive NBO therapy combined with endovascular mechanical thrombectomy, while the control group will receive a placebo air inhalation plus endovascular mechanical thrombectomy.

- OPENS2 (NCT04681651) [9]

A Randomized Controlled Trial Assessing the Efficacy and Safety of Normobaric Hyperoxia for Acute Ischemic Stroke Patients Undergoing Endovascular Treatment. This is a randomized controlled trial to assess the efficacy and safety of normobaric hyperoxia in acute ischemic stroke patients undergoing endovascular treatment. The study will enroll 280 participants who will be randomly assigned to either the experimental group, which will receive normobaric hyperoxia combined with endovascular mechanical thrombectomy, or the control group, which will receive an air placebo plus endovascular mechanical thrombectomy. The primary outcome measure is the modified Rankin Scale score 90 ± 14 days after randomization. The study started on 22 April 2021, and the estimated completion date is 1 December 2023 [9].

- PROOF (NCT03500939) [10]

Penumbral Rescue by Normobaric O=O Administration in Patients With Ischemic Stroke and Target Mismatch ProFile. The PROOF trial is a randomized controlled phase IIb clinical trial designed to examine the safety and efficacy of normobaric hyperoxygenation (NBHO) as a neuroprotective treatment for AIS caused by LVO and treated with MT. The trial enrolled 223 participants between August 2019 and August 2022. The study’s experimental arm receives NBHO in addition to standard care, while the control arm receives the standard care alone. The trial’s primary outcome measure is ischemic core growth from baseline to 24 h. The trial is single-blinded, with the outcome assessors being the only ones masked. We are currently waiting for the publication of the results [10].

### 3.2. Carbon Dioxide Targets

This systematic search identified four published studies and two ongoing studies.

#### 3.2.1. Published Studies

- Takahashi et al., 2013 [11]

This was a retrospective study that examined the association between the physiologic parameters—in particular, blood pressure and end tidal carbon dioxide (ETCO_2_)—and functional outcomes in 86 patients who underwent MT for AIS under GA. The primary outcome measured was the modified Rankin Scale (mRS) score at 90 days post-treatment. The results of the study showed that ETCO_2_ was significantly associated with the functional outcomes. Specifically, the ETCO_2_ levels at 60 and 90 min were significantly higher among patients with favorable outcomes compared to those with unfavorable outcomes. The study also found that the ETCO_2_ levels at 60 and 90 min remained highly associated with the functional outcomes even after controlling for other predictors of the outcomes. The study had several limitations, including being retrospective, having a small sample size, and being conducted at a single center. Additionally, the study did not measure the arterial partial pressure of CO_2_ (pCO_2_), which could have affected the results [11].

- Mundiyanapurath et al., 2016 [12]

This was an observational study with both retrospective and prospective phases on patients who underwent MT for AIS. The study aimed to identify the pulmonary and circulatory parameters as independent predictors of unfavorable outcomes. In the retrospective and pre-protocol phase, the study detected significant hypocapnia (ETCO_2_ less than 30 mmHg) in the 60 patients analyzed. In the prospective phase, characterized by the introduction of a protocol with the target values of ETCO_2_ and systolic blood pressure, the study identified that the incidences of severe hypocapnia were significantly reduced. The study also found that longer time in the ETCO_2_ range of 40–45 mm Hg was associated with an unfavorable outcome, but it was not an independent predictor. However, the study had limitations, such as retrospective data collection, significant heterogeneity in the groups of patients, and being a single-center, non-randomized study [12].

- Athiraman et al., 2018 [13]

This was a retrospective study that examined the records of 88 patients who received MT for AIS under GA to identify the factors associated with good functional outcomes. The outcomes measured were the mRS score at discharge and at 3 months. The authors stated that patients who had higher mean maximum ETCO_2_ (49 ± 8 vs. 45 ± 7 mmHg; *p* = 0.02) had good functional outcomes. After adjusting for age and NIHSS score, the study found that a higher maximum ETCO_2_ was an independent predictor for good outcomes at discharge. The study had several limitations, including being retrospective, having a small sample size, and being conducted at a single center [13].

- Parr et al., 2022 [14]

This was a retrospective study that aimed to investigate the effect of the intraprocedural mean ETCO_2_ levels on the core infarct expansion and neurologic outcome following MT for AIS in the anterior circulation under GA. The study included 58 consecutive patients who achieved successful recanalization and had CT perfusion, procedural ETCO_2_, and postoperative MRI data. The primary outcome was functional independence at 90 days. The results showed that patients with mean ETCO_2_ levels greater than 35 mmHg had higher rates of functional independence at 90 days. Although the data also showed that patients with mean ETCO_2_ greater than 35 mmHg had smaller final infarct volumes and percentages of penumbra that progressed to infarction, this did not reach statistical significance. The main limitations of this study were its retrospective design, the small sample size, and being performed in a single center [14].

#### 3.2.2. Ongoing Studies

- COMET AIS (NCT05051397) [15]

The CO_2_ Modulation in Endovascular Thrombectomy for Acute Ischemic Stroke (COMET-AIS) study aims to investigate the effect of moderate hypercapnia compared to normocapnia on cerebral vascular collaterality in patients with anterior circulation large vessel occlusion stroke who undergo thrombectomy under general anesthesia. The study will include 50 patients who meet the eligibility criteria and will be randomized to receive either moderate hypercapnia targeting a PaCO_2_ of 50 mmHg or normocapnia targeting a PaCO_2_ of 40 mmHg. The primary outcome measure is the ASITN cerebral vascular collaterality score before the reperfusion of the occluded vessel. The study is expected to be completed by October 2024 and will be conducted with double masking to ensure impartiality in the outcome assessment [15].

- SEACOAST 1 (NCT03737786) [16]

SEACOAST 1 is a prospective, randomized study aimed at comparing the clinical outcomes of two different forms of general anesthesia (GA) in patients with acute ischemic stroke due to anterior circulation large vessel occlusion (LVO) undergoing mechanical thrombectomy. The study will compare GA with normocarbia (PCO_2_ levels 40 mmHg ± 5%) vs. GA with mild hypercarbia (PCO_2_ levels 50 mmHg ± 5%), with a primary outcome of collateral robustness at catheter angiography and clinical efficacy as the secondary outcome. The study will enroll an estimated 90 patients who are eligible for MT at the University of California, Los Angeles Ronald Reagan Medical Center, and Santa Monica Medical Center. The primary outcome measure will be collateral circulation assessed through the Modified American Society of Interventional and Therapeutic Neuroradiology (ASITN) grading scale immediately prior to revascularization [16] (Table 1).

## 4. Discussion

Mechanical thrombectomy has become the standard treatment for large vessel occlusion in acute ischemic stroke [2]. With the increasing number of patients undergoing MT under general anesthesia and controlled ventilation, the need for specific ventilation strategies has arisen. This systematic review aimed to identify specific targets of arterial oxygenation and arterial carbon dioxide partial pressure that correlate with better neurological outcomes in patients undergoing MT for AIS.

### 4.1. Oxygenation Targets

The topic of oxygenation in stroke patients is still producing conflicting evidence and generating debate in the scientific community. While it is very well known that hypoxia in stroke patients leads to increasing morbidity and mortality [17], the role of hyperoxia has not yet been established. Severely ischemic brain tissues have a high oxygen demand, and increasing oxygen levels in these tissues may potentially delay the progression of the at-risk area (penumbra) toward infarction [18]. Various clinical studies have investigated the potential benefits of supplemental inhaled oxygen during acute stroke. However, despite encouraging findings in initial pilot studies [19,20], large-scale prospective trials have not demonstrated improved functional outcomes [21]. In the study published by Roffe in 2017, 8003 adults with AIS were enrolled and randomly assigned to receive continuous oxygen for 72 h, nocturnal oxygen for 3 nights, or oxygen only if clinically indicated. The primary outcome was mRS at 90 days. The results showed that no subgroup of patients was identified to benefit from oxygen supplementation. Therefore, the study concluded that prophylactic low-dose oxygen supplementation is not effective in reducing death or disability in non-hypoxic patients with acute stroke [21]. Controversy surrounds the indiscriminate delivery of oxygen, as it may pose risks to non-hypoxic tissues, including the formation of free oxygen radicals, pulmonary toxicity, and adverse effects on the vascular tone and blood pressure mediated by oxygen [22]. Furthermore, the clinical trial with the identifier NCT00414726, also known as the Normobaric Oxygen Therapy in Acute Ischemic Stroke Trial, was terminated prematurely after enrolling 85 out of 240 patients due to the increased mortality observed in the high-flow oxygen group. It is important to note that the majority of the deaths occurred following the early withdrawal of life support measures [23]. 

In this SR on ventilation targets for patients with AIS undergoing MT, we did not find any relevant studies investigating intraprocedural oxygenation targets and their correlation with neurological outcomes. However, we identified two published studies that examined early postoperative oxygenation targets in this patient population, as well as three ongoing studies relevant to these intraprocedural goals. Interestingly, these two published studies provided conflicting evidence. Cheng et al. reported that supplemental high-flow normobaric oxygen therapy was associated with a higher proportion of patients showing improved functional outcomes [7]. Conversely, López et al. found that higher levels of arterial oxygen partial pressure (paO_2_) were predominantly associated with worse functional outcomes in these patients. The latter study concluded that admission paO_2_ levels above 120 mmHg were linked to poorer functional outcomes in ischemic stroke patients undergoing MT [6]. It is important to note that both studies had limitations, including small sample sizes and being conducted at single centers.

Currently, there are three ongoing trials (NBOL, NCT05039697; OPENS2, NCT04681651; and PROOF, NCT03500939) that have the potential to provide further insights into whether there is an optimal level of oxygenation during MT for acute ischemic stroke that leads to improved neurological outcomes.

### 4.2. Carbon Dioxide Targets

Carbon dioxide possesses potent cerebral vasodilatory properties, with a direct correlation observed between the cerebral blood flow (CBF) and arterial carbon dioxide concentrations (PaCO_2_) within the range of 2.5 to 8.0 kPa (20 to 60 mm Hg) [24,25]. This effect is believed to be mediated by alterations in the extracellular pH resulting from carbon dioxide-related changes [26]. The responsiveness of cerebrovascular resistance and CBF to changes in PaCO_2_ is referred to as cerebrovascular reactivity to carbon dioxide. Therefore, carbon dioxide can act as a potent cerebral vasodilator that has the potential to influence outcomes in ischemic stroke cases. By acting as a vasodilator, CO_2_ can enhance the collateral circulation to at-risk brain tissue during acute ischemic stroke, thus slowing down the progression of the ischemic penumbra towards complete infarction [27]. Animal experiments have previously demonstrated the beneficial effects of elevated CO_2_ levels in hypoxic ischemic brain injury [28]. On the other hand, induced hypocapnia by hyperventilation is a common practice in neuroanaesthesia when life-saving acute control of intracranial hypertension is needed. Prolonged hypocapnia can produce cerebral vasoconstriction and potential cerebral ischemia and injury [29].

Regarding the influence of carbon dioxide on the neurological outcomes in patients undergoing mechanical thrombectomy for acute ischemic stroke, our systematic review identified four published papers and two ongoing trials. Although the available evidence from the published papers was limited, it suggested a potential association between intraoperative hypocapnia during MT and poorer neurological outcomes. However, due to the retrospective nature and small sample sizes of these studies, they were unable to identify a specific target that provided better outcomes. Therefore, it is challenging to generate clinical recommendations based on this weak evidence.

The ongoing trials COMET AIS (NCT05051397) and SEACOAST 1 (NCT03737786) have implemented robust methodologies to investigate the variable pCO_2_ during MT and its correlation with the intraoperative collaterality levels and subsequent neurological outcomes. Once these studies are completed and published, we can expect more substantial evidence to support its clinical practice.

## 5. Conclusions

Given the anticipated scarcity of scientific evidence on this topic, this SR adopted broad inclusion criteria, encompassing all original human studies without excluding any methodologies, in order to capture as much available evidence as possible. Consequently, the analysis encompassed a heterogeneous set of studies. Therefore, this SR identified limited evidence regarding specific ventilation strategies in patients undergoing MT for acute ischemic stroke.

Further studies, particularly randomized controlled trials, are needed to determine the optimal oxygenation and carbon dioxide targets during MT for acute ischemic stroke and their potential impacts on neurological outcomes.

In conclusion, based on the currently available evidence and in line with the American Heart Association (AHA) guidelines for acute ischemic stroke management, it seems reasonable to administer additional oxygen only if a patient’s SpO_2_ falls below 94%, taking caution to titrate the PaO_2_ levels carefully to prevent both hypoxemia and excessive hyperoxemia [30,31]. Furthermore, considering the discussed evidence, it would be advisable to avoid hyperventilation and hypocapnia in patients receiving mechanical ventilation during MT for AIS.

## Figures and Tables

**Table 1:** Description of the characteristics of the included studies.

First Author Year Reference	Study Design Number of Patients (N)	Primary Endpoint	Secondary Endpoint	Key Message
López et al. 2019 [6]	Prospective. *N* = 333 pO_2_ > 120 mmHg: *n* = 119 pO_2_ < 120 mmHg: *n* = 214	mRS at 90 days	ICU length of stay and NIHSS at 24 h.	Higher mRS score and mortality after 90 days in the hyperoxia group. NIHSS values at 24 h and the length of stay in ICU were significantly higher in the hyperoxia group.
Cheng et al. 2021 [7]	RCT. *N* = 175 NBO: *n* = 88 Control group: *n* = 87	mRS score at 90 days	NIHSS at 24 h, infarct volume, mortality, symptomatic ICH, fatal ICH, pneumonia, urinary infection, seizures	Better functional outcomes at 90 days in NBO group than in the control group. The mortality at 90 days was lower in the NBO group than in the control group. Reduction of infarct volume in the NBO group. No differences in the rate of symptomatic ICH, pneumonia, urinary infection, and seizures between the groups.
Takahashi et al. 2014 [11]	Retrospective. *N* = 86	mRS at 90 days		Patients with better functional outcomes had higher EtCO_2_ at 60 and 90 min in comparison to those with unfavourable outcomes (mRS 4–6). There was no association between BP and functional outcomes at 90 days.
Mundiyanapurath et al. 2016 [12]	Observational study with retrospective and prospective phases *N* = 124 Retrospective group: *n* = 60 Prospective group: *n* = 64	mRS at 90 days		Longer duration of EtCO_2_ values within 40–45 mmHg and a higher cumulative dose of norepinephrine were associated with an unfavorable outcome (mRS > 2).
Athiraman et al. 2018 [13]	Retrospective. *N* = 88	mRS at discharge	mRS at 90 days	Patients with a higher mean maximum ETCO_2_ (49 ± 8 vs. 45 ± 7 mmHg) had good functional outcomes. Independent predictors of good outcomes were a higher maximum EtCO_2_ and extubation after MT.
Parr et al. 2022 [14]	Retrospective. *N* = 88	mRS at 90 days	Ischemic penumbra and infarct volume	Procedural ETCO_2_ exceeding 35 mmHg had better functional outcomes. No statistical significance in salvaging more penumbra and smaller final infarcts when ETCO_2_ exceeded 35 mmHg.
